# An Umbrella Review of Systematic Reviews and Meta-Analyses Evaluating the Success Rate of Prosthetic Restorations on Endodontically Treated Teeth

**DOI:** 10.1155/2022/4748291

**Published:** 2022-02-22

**Authors:** Amirhossein Fathi, Behnaz Ebadian, Sara Nasrollahi Dezaki, Nahal Mardasi, Ramin Mosharraf, Sabire Isler, Shiva Sadat Tabatabaei

**Affiliations:** ^1^Department of Prosthodontics, Dental Materials Research Center, Dental Research Institute, School of Dentistry, Isfahan University of Medical Sciences, Isfahan, Iran; ^2^Dental Implants Research Center, Department of Prosthodontics, School of Dentistry, Isfahan University of Medical Sciences, Isfahan, Iran; ^3^Dental Students' Research Committee, School of Dentistry, Isfahan University of Medical Sciences, Isfahan, Iran; ^4^Department of Prosthodontics, Ahvaz Jundishapur University of Medical Sciences, Ahvaz, Iran; ^5^Department of Prosthodontics, Faculty of Dentistry, Istanbul University, Istanbul, Turkey

## Abstract

**Materials and Methods:**

The electronic search was conducted in the MEDLINE/PubMed, Cochrane, and Google Scholar databases until November 2020, regardless of language limitations. The inclusion criterion was as follows: S/M-R regarding prosthetic restorations in endodontically treated teeth. Three qualified researchers evaluated the inclusion criteria and bias risk. The fourth investigator was referred to when facing any doubtfulness.

**Results:**

From 43 achieved S/M-R, 14 studies were selected for this inquiry. Primary extracted information included success rate, survival rate, and postendodontic failure rate. Five S/M-R had a moderate risk of bias, and nine S/M-R had a low risk of bias and were considered strong clinical evidence in this examination. According to the low-risk reports, the success rate of fiber posts was higher than that of metal posts; the rate of root fracture in metallic and fiber posts was alike; the failure rate for fiber posts was comparable to fixed partial dentures or single crowns; the construction of endocrowns was likely to perform better than intracanal posts, composite resin, or inlay/onlay restorations.

**Conclusion:**

It appears that with practice and experience, deciding which type of restoration to choose changes. In dental restorations associated with root canal therapy, the single crowns are likely to be a proper option. Nevertheless, due to the heterogeneity of the studies, more clinical assessments are required to achieve more specific findings in this field.

## 1. Introduction

Endodontic therapy is a routine and standard dental treatment [1–7]. Through endodontic therapy, tooth material is unavoidably sacrificed, and the tooth is weakened [8, 9]. It is thereby apparent that ET teeth require restoration [10, 11]. Determining the proper restoration for an endodontically treated tooth is associated with the number of vital teeth, anatomical situation, occlusal pressure, and restorative and aesthetic necessities of the tooth [12]. In general, benefits can be achieved through both traditional direct restorations and prosthetic restorations such as crowns, fixed partial dentures, removable partial dentures, and mixed removable-fixed prostheses, with or without postplacement.

The application of posts has been widely discussed in dentistry for a long time, and they are commonly suggested when the amount of remaining hard tissue is crucial [13, 14]. Popular postsystems consist of both cast and prefabricated posts with a broad order of substances. The use of different posts requires applying particular principles [15]. Recently, fiber-reinforced posts have been introduced in addition to traditional metal posts to preserve teeth with a small amount of residual structure. Since mechanical properties of the whole system, including post, cement, and dentine should be homogenous, engaging in fiber posts cemented and reconstructed by composite resin material is likely to ensure a good performance [16].

When it is impossible to use implants, removable or fixed dentures, restoring ET teeth is more critical. The results of a systematic examination [17] showed that the retention, satisfaction, and cost-effectiveness of restored teeth with a single crown or implant are higher than fixed or removable dentures. However, other systematic studies have found no significant difference between the survival rate of restored ET teeth with single crowns and implants [18].

Based on several current systematic reviews [19], endodontically treated (ET) teeth present a predicted survival rate of 87% within eight to ten years. Researchers evaluated cases of monitoring teeth after both first endodontic therapy and retreatment, excluding periapical surgery treatments. Different epidemic-related studies [20], where a substantial cohort was examined (over one million cases), obtained a survival rate of 97% in ET teeth after eight years.

One of the most influential and determining factors for the success of endodontic therapy is whether a periapical injury exists former to practice [21–25]. Other circumscribing parameters contain the amount of the root filling in association with the root crown [22–24, 26], pulp status before therapy [23, 24], postendodontic coronal restoration [20, 25, 27, 28], and proximal contacts [19]. It is assumed that molars exhibit significantly lower survival rates than other teeth; this theory has been confirmed by former cohort investigations [29, 30]. Others have been incapable of notably relating particular teeth to the durability of treatment [31]. More elements have also been examined in previous reports. However, confirmation of their influence on the survival rate of ET teeth is weak or uncertain. These factors include age [24, 25, 31], the kind of post [32–35], root filling mass [24, 27], and the number of sessions until the end of endodontic therapy [36, 37].

Despite the abundance of S/M-R in this area, a lack of consensus is seen among specialists [38, 39] and information gaps cause failures in clinical practice. Therefore, valid scientific documentation is required to make a proper decision. Considering the lack of compliance among studies on a particular technique or methodology and since scientific studies require impartiality, Cochrane proposed a new kind of study called the S/M review, in which findings from multiple S/M-R are combined into one text to increase confidence in decision-making by comparing scientific data [40, 41]. The purpose of this overview was to find S/M-R determining ET teeth restoration success and evaluate the quality level of studies on the success of ET teeth treatment methods.

## 2. Materials and Methods

### 2.1. Search Strategy

Electronic search was conducted in PubMed/MEDLINE, Cochrane, and Google scholar databases until November 2020 without language limitation. The research included S/M-R and their references that examined the success of prosthetic procedures in ET teeth.

The PICO inquiry (population, intervention, comparison, and the outcome) was followed. The population included teeth that have received root canal treatments. The intervention was providing restorations for ET teeth. There was no control; hence, no comparison was performed. The outcome contained the survival rate and failure rate of restored ET teeth (Table 1).

This review study was conducted using the guidance on preferred reporting elements for systematic reviews and meta-analyses [42]. The AMSAR2 [43] method was also used for calculating the risk of S/M-R bias. Selected keywords included “prosthetic restorations” and “endodontically treated teeth.”

Inclusion and exclusion criteria in S/M-R screening.

Inclusion criteria were as follows:

1. S/M-R studies
2. Studies in English language only
3. Evaluating the success/failure rate of prosthetic restorations in endodontically treated teeth.

Exclusion criteria included duplicate reviews, comments, and editorials.

Full texts of studies that met our inclusion criteria were received, and these studies were considered eligible for our study.

### 2.2. Data Collection Process

The data were collected by three independent researchers who had already received adequate training in this field (kappa = 1.0). Required information such as prosthesis type, success, and survival rate was extracted from each systematic study. If there were any inconsistencies or ambiguities, the matter was resolved through discussion. If the issue was not resolved, the fourth investigator was asked to provide assistance.

### 2.3. Bias Risk Assessment

Based on the risk of bias assessment [43], 16 questions were used to evaluate the quality and bias of the S/M-R (Table 2). In the end, each article received a score that indicated the risk of bias in that study. If eight to eleven positive responses were received, the risk of bias was low; if four to seven questions were answered positive, the risk of bias was moderate; and if fewer than three questions received a positive response, the risk of bias was assessed as high [40]. The assessment was conducted by two qualified investigators (kappa = 0.9). If there were any inconsistencies or ambiguities, the matter was resolved through discussion. If the ambiguities were not cleared up, the third investigator was asked to assist.

## 3. Results

### 3.1. Screening of S/M-R

**Figure 1:**
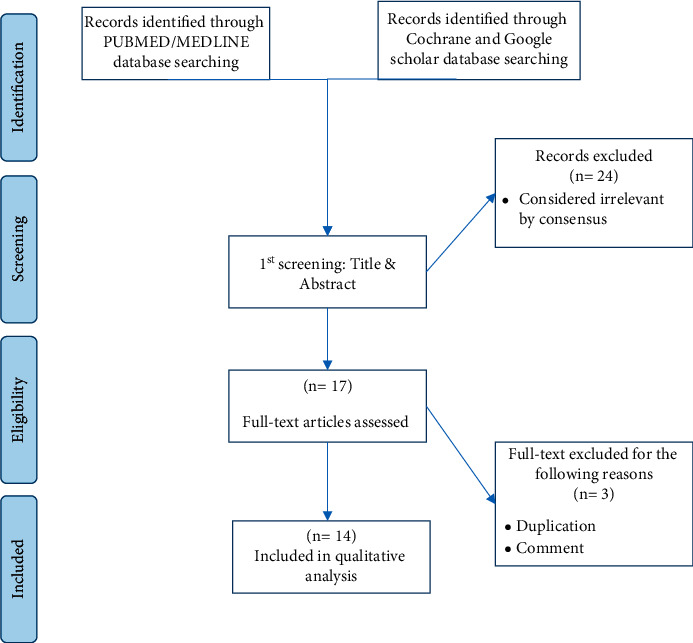
Flowcharts for the studies were identified, displayed, and included in the study.

In the initial search, 43 articles were found, of which 36 articles were obtained by PubMed/MEDLINE, five articles by Cochrane, and two studies by manual search. Then, after reviewing the title, abstract, and inclusion/exclusion criteria, 17 studies were selected. Finally, based on the full text of the articles, 14 S/M-R [44–57] were eventually included in our study (Figure 1). We collected the S/M-R in three parts: prosthesis, success rate, and ET teeth failure rate. The overall number of studies that were analyzed in selected reviews and therefore included in our study was 118.

### 3.2. Risk of Bias Assessment

We applied the Assessment of Multiple Systematic Reviews (AMSR2) tool to measure the risk of bias used for various studies. Based on the number of correct responses, the level of bias was reported as high, medium, or low (Table 2). In this study, the risk of bias was moderate [including five S/M-R: [44, 45, 47, 56, 57]] and low [including nine S/M-R: [46, 48–55]]. In addition, 41.5% of all surveys represented low-risk S/M-R (Table 1). Reliable clinical evidence was expected from S/M-R studies with a low risk of bias.

### 3.3. Characteristics of Systematic Reviews

General information of each S/M-R is presented in Table 3. They include authors and year of publication, number and type of studies, type of analysis, research period, interventions, outcomes, risk of bias, and main results.

### 3.4. General Sample Analysis

- The success rate of prosthetic restorations on ET teeth: In three S/M-R [44, 51, 54], containing a total number of 22 studies (RCTs [1], in vitro studies [12], clinical trials [6], prospective studies [4], and retrospective studies [1]), the success rate was compared. In one study [44], the five-year success rate for endocrowns and conventional crowns was about 77% and 94%, respectively. Sedrez-Porto et al. [51] and Ploumaki et al. [54] also reported success rates of 92%, 79%, and 66% for single crowns, and fixed and removable prostheses, respectively.
- The success rate of single crowns in ET teeth restored with or without posts: Three S/M-R [45, 48, 54] including 34 studies were surveyed. According to Ploumaki et al. [54], the success rates of single crowns on teeth without posts, with posts, with cast post and cores, and with prefabricated posts were 94%, 92%, 93%, and 94%, respectively. Sarkis-Onofre et al. [48] reported the success rate of elastic posts from 71.8 to 100%. Girotto et al. [45] stated that the most frequently used posts were firstly prefabricated and secondly metal posts with rates of 45.8% and 16.7%, respectively. They mentioned time and training as factors affecting the decision of choosing prefabricated or metal posts.
- Survival rate of single crowns on ET teeth: eight S/M-R articles [44, 46–49, 53, 56, 57], listed in Table 3, including 48 RCT, 13 in vitro, 10 clinical trials, and 2 prospective studies, provided information on survival. Based on the study performed by Al-Dabbagh [44], the overall 5-year survival rates for endocrowns and conventional crowns were 91.4% and 98.3%, respectively. In the study performed by Suksaphar et al. [49], the survival rate of crowns was 94%, and the composite resin survival rate was 91%. In addition, according to the study of Stavropoulou and Koidis [56], the 10-year survival rates for crowns and direct restorations were 81% and 63%, respectively. Suksaphar et al. [49] reported that the survival rate of composite resin or crowns against fracture was nearly the same. Wang et al. [46] concluded that the survival rate of fiber posts was significantly higher than metal posts. They found that for root treatment with more than two crowned walls, the medium-term survival rate of fiber posts was higher than metal posts. Figueiredo et al. [53] reported that the survival rates of metal posts and fiber posts were 90% and 83.9%, respectively. In addition, the survival rate of cast post and cores in the study by Heydecke and Peters [57] ranged from 87.2% to 88.1%. Naumann et al. [47] also stated in their study that ferrule increases the survival rate of endodontic-treated teeth by preserving cavity walls. According to Sarkis-Onofre et al. [48], teeth without ferrule also showed a higher variation in the survival rate (0%–97%) compared to teeth with ferrule.
- Failure rate: The six S/M-R [46, 50, 51, 53, 54, 57] listed in Table 3 contained 48 studies including RTCs [14], in vitro studies [11], clinical trials [13], prospective studies [3], and cohorts [7]. They reported failure rates in their systematic reviews. According to Wang et al. [46], the success rate, postdebonding rate, or root fracture rate between fiber posts and metal posts were not significantly different. Moreover, according to the study by Figueiredo et al. [53], the rate of root fractures in metal and fiber posts was similar. Furthermore, the rate of root fractures in prefabricated metal posts and carbon fiber was twice as high as that of metal posts and fiberglass. In the review performed by Heydecke and Peters [57], the failure rate between direct posts and direct post and cores was not significantly different. According to the study performed by Sorrentino et al. [50], the most frequent failures in single crowns and fixed prostheses were caused by the separation of the fiber post, lack of single crown retention, and marginal clefts. In Sedrez-Porto et al. [51] study, the rate of failure in endocrowns was reported higher than that of conventional methods. Ploumaki et al. [54] also reported postdebonding as the most common cause of failure.

## 4. Discussion

Due to controversy among studies on the success rate of endodontically treated teeth, the purpose of this umbrella review was to compare the clinical evidence for the success and failure rates of restorations in endodontically treated teeth. Targets included fixed/removable prostheses and posts and the rate of success and failure in them. Data from 14 systematic reviews [44–57], which included 118 studies and more than 10971 samples altogether, were categorized based on the type of restoration.

In 22 of the 118 studies, successful single crowns on ET teeth were reported exclusively [58–60]. Furthermore, in 34 studies, the success rate of single crowns on ET teeth was compared with or without posts. The five-year success rate of the endo-crown system was 94% [1, 3]. Eventually, the survival rate of ET teeth was associated with the remaining crown structures and the type of restorative material. Both of these play an important role in increasing the chance of long-term dental survival. Based on clinical studies, a single crown is the best treatment for ET teeth. However, high-quality clinical evidence on this subject is required due to the limited number of data available.

Within the systematic review studies, the results from four studies were about fixed and removable prostheses. Thirty-four studies reported postrestoration success. The success rate for ET teeth [58] after six years was 94–92%. Furthermore, the success rate for fixed and removable prostheses was 78% and 66%, respectively. In general, single crowns perform better than other prosthetic restorations [28, 61]. This is because the dental stresses in fixed and removable dentures are more than single crowns [28]. Besides, removable dentures should also be reinforced with posts [13, 28]. According to the results of the review studies, the success rate for prefabricated posts was higher than cast/core posts. Clinically, however, dentists engage in cast/core posts when dealing with moderate to high tissue loss. Findings from various studies show that nonmetallic fiber posts work better than metal posts [55, 62, 63]. However, the overall evidence in this area is relatively weak and should be interpreted more accurately.

One of the tools to assess the quality and bias of studies is the AMSTAR tool, which is designed based on responses to a standard set of questions. It is necessary to correctly interpret the information to use quality measurement tools and determine the S/M-R proficiency level. However, during this assessment, the Funding Sources parameter in the text was not indicated. Therefore, more attention to this issue is recommended in articles. Despite the limitations of this study, we evaluated the results of several systematic reviews comprehensively and tried to eliminate some controversies among their results. Nevertheless, more investigation is recommended in this regard to draw a more reliable conclusion for clinicians.

## 5. Conclusion

It appears that one of the most reliable ways to restore ET teeth is to apply single crowns and endocrowns. However, more consistent studies are required to present the reported findings more confidently. Even considering the potential for bias, the level of evidence available for the use of this clinical method is high.

## Figures and Tables

**Table 1:** PICO strategy.

PICO inquiry	Description
Population	Teeth receiving root canal treatments
Intervention	Restoring endodontically treated teeth
Comparison	No comparison was determined
Outcome	Survival rate and failure rate of restored ET teeth

**Table 2:** The assessment of multiple systematic reviews (AMSTAR2).

References	Question & inclusion	Protocol	Study design	Comprehensive search	Study selection	Data exclusion	Exclude study justification	Include study details	Risk of bias (RoB)	Funding sources	Statistical methods	RoB on meta- analysis	RoB in individual studies	Explanation for heterogeneity	Publication bias	Conflict of interest	Results according to number of yeses	Criteria for AMSTAR analysis according to positives answers	Review quality
	1	2	3	4	5	6	7	8	9	10	11	12	13	14	15	16	-		
[44]	Y	PY	Y	PY	Y	N	N	PY	N	N	Y	N	Y	N	N	N	5	Moderate risk	Low
[45]	Y	PY	Y	PY	Y	N	N	PY	PY	N	N	Y	Y	N	N	N	5	Moderate risk	Low
[46]	Y	Y	Y	Y	Y	Y	Y	N	PY	N	Y	Y	Y	Y	Y	N	12.5	Low risk	High
[47]	Y	PY	Y	Y	Y	Y	N	PY	PY	N	NMC	NMC	N	N	NMC	Y	6.5	Moderate risk	Moderate
[48]	Y	Y	Y	Y	Y	Y	N	PY	Y	N	NMC	NMC	Y	Y	NMC	Y	10.5	Low risk	High
[49]	Y	PY	Y	PY	Y	Y	Y	PY	N	N	NMC	NMC	N	N	NMC	Y	7.5	Low risk	Moderate
[50]	Y	PY	Y	PY	Y	N	Y	PY	Y	N	NMC	NMC	N	N	NMC	Y	7.5	Low risk	Moderate
[51]	Y	Y	Y	PY	Y	N	N	PY	Y	N	Y	Y	Y	N	Y	N	10	Low risk	High
[52]	Y	Y	Y	PY	Y	N	Y	PY	Y	Y	Y	Y	Y	Y	Y	Y	14	Low risk	High
[53]	Y	Y	Y	Y	Y	N	N	PY	Y	N	Y	Y	Y	Y	Y	Y	12.5	Low risk	High
[54]	Y	Y	Y	PY	Y	N	Y	Y	N	N	Y	N	N	Y	N	Y	9.5	Low risk	Moderate
[55]	Y	Y	Y	Y	Y	N	Y	Y	Y	N	Y	Y	N	Y	Y	N	12	Low risk	High
[56]	Y	PY	Y	PY	Y	Y	N	Y	N	N	N	N	N	N	N	N	6	Moderate risk	Moderate
[57]	Y	PY	Y	PY	N	N	N	PY	N	N	N	N	N	N	N	N	3.5	Moderate risk	low

Y: yes; N: no; PY: partial yes; NMC: no meta-analysis conducted. Overall methodological quality: low: 0–5; moderate: 5–10; high: 10–16. Criteria for AMSTAR analysis according to positive answers: low risk (8–11), moderate risk (4–7), and high risk (≤3).

**Table 3:** Baseline characteristics of systematic reviews assessing the prosthetic restorations on endodontically treated teeth.

Author (year)	Types/no. of studies included	Method of analysis	Search period	Interventions	Overall number of samples (restored teeth)	Outcomes accessed	Risk of bias	Main results
Al-Dabbagh, 2020 [44]	3 clinical trial/7 in vitro	SR/MA	Up to June 2019	Evaluation of survival and success of endocrowns in ET teeth restoration	376	Restoration materials, restoration methods, survival rate, success rate, failure rate	Moderate	Endocrowns is a promising restorative option for ET posterior teeth

Girotto et al., 2020 [45]	25	SR/MA	Up to Nov 2019	Preferences of dentists and students in choosing the type of restoration in ET teeth	600	Type of posts Prefabricated posts Cast metal posts	Moderate	Restorative preferences related to posts have changed over time, from cast posts to prefabricated ones or the use of both posts. They seem to be influenced by experience and postgraduate training

Wang et al., 2019 [46]	4 RCTs	SR/MA	Up to Jan 2018	Fiber posts vs. metal posts for restoration	223	Fiber posts survival rate Metal posts survival rate Success rates Post debonding rates Root fracture rates	Low	Fiber posts displayed higher medium-term overall survival rates than metal posts when used to restore ET teeth with no more than two coronal walls remaining.

Naumann et al., 2018 [47]	7 RCTs 1 prospective	SR	June 2017	Postendodontic treatment using posts with or without ferrule	1530	Failure rates of post/core complexes with or without ferrule support tooth and/or restoration survival	Moderate	Ferrule effect and maintaining cavity walls are the predominant factors concerning tooth and restoration survival of ET teeth

Sarkis-Onofre et al., 2017 [48]	9 RCTs	SR	2004 to 2013	Influence of the number of remaining coronal walls, the use or disuse of posts, and their type	1526	Post- or crown cementation endodontic failure crown/postfracture crown dislodgements postdebonding rates clinical/radiographic examination	Low	Should focus on the maintenance of the coronal structure

Suksaphar et al., 2017 [49]	1 RCTs 1 Prospective 1 Retrospective	SR	1980 to 2016	Crowns or resin composite for posterior teeth restored	116	Survival rate against fracture	Low	The survival rates against the fracture of ET posterior teeth restored with crowns or resin composites were not significantly different in the teeth with minimum to moderate loss of tooth structure

Sorrentino et al., 2016 [50]	4 RCTs	SR	Up to 2015	Fiber posts and single crowns or fixed dental prostheses for restoration	117	Failure rates of fiber posts prosthetic restorations	Low	A correlation between the failure rates of fiber posts and the type of prosthetic restorations, just like SCs and FDPs, cannot be found to date
Sedrez-Porto et al., 2016 [51]	3 Clinical trial 5 In vitro	SR/M-A	Up to February 2016	Endocrown compared to conventional treatments (intraradicular posts, direct composite resin, and inlay/onlay). For restorations	—	Fracture strength endocrown restorations conventional restorations	Low	Endocrowns may perform similarly or better than the conventional treatments using intraarticular posts, direct composite resin, or inlay/onlay restorations

Sequeira‐Byron et al., 2015 [52]	I RCTs	SR/MA	Up to March 2015	Single crowns versus conventional fillings	—	Catastrophic failure of restoration, noncatastrophic failure of restoration, noncatastrophic failure of post	Low	There is insufficient evidence to assess the effects of crowns compared to conventional fillings to restore root-filled teeth

Figueiredo et al., 2015 [53]	7 RCTs 7 Cohort	SR/MA	Up to January 2014	Incidence rate related to the use of metal posts was higher than that of fiber posts	3202	Metal-based posts survival rate fiber-reinforced posts survival rate catastrophic failures	Low	Results did not show significant differences for root fracture incidence between metal and fiber posts

Ploumaki et al., 2013 [54]	1 RCTs 3 Prospective	SR/MA	Up to June 2012	The success rates of prosthetic restorations on endodontically treated teeth	1206	Success rate of single crowns success rates of crowns over cast post and core success rates of crowns over prefabricated posts	Low	The results of this systematic review should be interpreted with caution

Bolla et al., 2007 [55]	2 RCTs	SR/MA	CENTRAL to 2005 MEDLINE to September 2005/Scopus to December 2004 EMBASE to D es 2004	Root canal posts for the restoration	317	Loss of retention, postfracture root fracture	Low	It is not specified which type of post and core system should be used when two or three dentine walls remain

Stavropoulou and Koidis, 2007 [56]	10 RCTs	SR/MA	1960 to 2006	Placement of a crown is associated with improved (long term) survival of root canal treated teeth	—	Survival of RCT restored with crowns survival of RCT with direct restorations	Moderate	RCTs restored with crowns show an acceptable long-term survival of 10 years, while direct restorations have an excellent survival only for a short period.

Heydecke and Peters 2002 [57]	10 Clinical trial 6 In vitro	SR/MA	1995 and 2000	Single-rooted teeth with cast or direct posts and cores	1758	Load-to-failure Cast post and core failure Direct post and core failure	Moderate	No significant difference between cast and direct posts and cores

SR: systematic review;/MA: meta-analysis; RCTs: randomized clinical trials.
